# Hyaluronan-Containing Injectable Magnesium–Calcium Phosphate Cements Demonstrated Improved Performance, Cytocompatibility, and Ability to Support Osteogenic Differentiation In Vitro

**DOI:** 10.3390/ijms26146624

**Published:** 2025-07-10

**Authors:** Natalia S. Sergeeva, Polina A. Krokhicheva, Irina K. Sviridova, Margarita A. Goldberg, Dinara R. Khayrutdinova, Suraya A. Akhmedova, Valentina A. Kirsanova, Olga S. Antonova, Alexander S. Fomin, Ivan V. Mikheev, Aleksander V. Leonov, Pavel A. Karalkin, Sergey A. Rodionov, Sergey M. Barinov, Vladimir S. Komlev, Andrey D. Kaprin

**Affiliations:** 1 P.A. Herzen Moscow Research Oncology Institute, Branch of Federal State Budgetary Institution “National Medical Research Radiological Centre”, Ministry of Health of the Russian Federation, 2nd Botkinsky Pass. 3, 125284 Moscow, Russia; prognoz.01@mail.ru (N.S.S.); tagieva58@mail.ru (S.A.A.); kirik-57@mail.ru (V.A.K.); pkaralkin@gmail.com (P.A.K.); rodionov_085@mail.ru (S.A.R.); kaprin@mail.ru (A.D.K.); 2 Baikov Institute of Metallurgy and Materials Science, Russian Academy of Sciences, 119334 Moscow, Russia; polinariakroh@gmail.com (P.A.K.); mgoldberg@imet.ac.ru (M.A.G.); dvdr@list.ru (D.R.K.); oantonova@imet.ac.ru (O.S.A.); afomin@imet.ac.ru (A.S.F.); barinov_s@mail.ru (S.M.B.); 3 Faculty of Chemistry, M.V. Lomonosov Moscow State University, 119991 Moscow, Russia; mikheev.ivan@gmail.com (I.V.M.); aleonov49@gmail.com (A.V.L.); 4 L.L. Levshin Institute of Cluster Oncology, I.M. Sechenov First Moscow State Medical University, Trubetskaya 8, Build 2, 119991 Moscow, Russia; 5 Federal State Budgetary Institution, “N.N. Priorov National Medical Research Center of Traumatology and Orthopedics”, The Ministry of Health of the Russian Federation, Priorova Street 10, 127299 Moscow, Russia; 6 FSBI National Medical Research Radiological Centre, Ministry of Health of the Russian Federation, Koroleva Street 4, 249036 Obninsk, Russia; 7 Head of the Department of Oncology and Roentgenology, RUDN University, Miklukho-Maklay Street 6, 117198 Moscow, Russia

**Keywords:** magnesium phosphate bone cement, physicochemical properties, bioactivity in vitro, cytotoxicity, cytocompatibility, gene expression

## Abstract

Due to their biocompatibility, biodegradability, injectability, and self-setting properties, calcium–magnesium phosphate cements (MCPCs) have proven to be effective biomaterials for bone defect filling. Two types of MCPC powders based on the magnesium whitlockite or stanfieldite phases with MgO with different magnesium contents (20 and 60%) were synthesised. The effects of magnesium ions (Mg^2+^) on functional properties such as setting time, temperature, mechanical strength, injectability, cohesion, and in vitro degradation kinetics, as well as cytocompatibility in the MG-63 cell line and the osteogenic differentiation of BM hMSCs in vitro, were analysed. The introduction of NaHA into the cement liquid results in an increase in injectability of up to 83%, provides a compressive strength of up to 22 MPa, and shows a reasonable setting time of about 20 min without an exothermic reaction. These cements had the ability to support MG-63 cell adhesion, proliferation, and spread and the osteogenic differentiation of BM hMSCs in vitro, stimulating *ALPL*, *SP7*, and *RUNX2* gene expression and *ALPL* production. The combination of the studied physicochemical and biological properties of the developed cement compositions characterises them as bioactive, cytocompatible, and promising biomaterials for bone defect reconstruction.

## 1. Introduction

Life expectancy and the associated increase in the number of older people are on the rise. The World Health Organization estimates that while people over 60 years of age accounted for 12 percent of the world’s population in 2015, in 2030, they will account for over 15 percent (or 1.4 billion people), and in 2050, they will account for 22 percent (or 2.1 billion people) [1]. The increase in life expectancy has naturally led to a significant increase in the number of patients with pathological processes in bone tissue requiring surgical interventions that reconstruct bone defects of different natures [2,3,4,5]. Thus, according to Wenhao Wang and Kelvin W.K. Yeung, more than two million operations involving bone autogeneic and allogeneic tissues are performed annually worldwide, making it the second most frequent tissue transplantation after blood transfusion [6,7,8,9,10,11]. The autologous and allogeneic grafts are united by the presence of cytocompatibility and biocompatibility. At the same time, they differ in biodegradation rates and in their expression of osteoconductive and osteoinductive potencies, which provide the ability to serve as scaffolds supporting new bone growth and stimulating stem cells or undifferentiated mesenchymal cells to differentiate into bone-forming cells. These differences affect the quality of osteointegration, i.e., they have both obvious advantages and limitations in use [12,13,14]. The necessity of opening a second operating field, limitations in obtaining a sufficient amount of material, and possible subsequent complications in the donor area are obstacles to the widespread clinical use of autologous bone grafts [14,15]. Clinicians associate allogeneic implants with the risks of transferring infectious agents, lower mechanical strength, and the biodegradation rate of allogeneic bone, which is inconsistent with the rate of new bone tissue formation [16,17].

All of the above dictate the necessity of using synthetic biomaterials for bone defect replacement/reconstruction. The most commonly used bioresorbable osteoplastic materials are calcium phosphate bioceramics [18,19,20], natural and synthetic polymers [10,21,22], composite biomaterials [23,24], and bone cements [25,26,27,28]. In the last decade, there has been an increase in the use of injectable bone cements in bone reconstructive and plastic surgery [29,30,31]. This is due to their ability to fill bone defects during injection from a syringe, including defects of the most complex configurations; tight adherence to the mother bed; the presence of osteoconductive and osteointegrative properties; and sufficient mechanical strength [31]. In this regard, several additional requirements for modern bone cements have been formulated: injectability, the absence of an exothermic reaction when the cement powder and setting liquid are combined, a setting time appropriate for surgery, excellent anti-washout ability to prevent the cement from crumbling in biological fluids, physiological or close to physiological pH value of the set cement, and the biodegradation ability of set cement with further de novo bone tissue formation in the implantation area [32].

Doping with additional elements, particularly magnesium, in calcium phosphate cements (CPCs) powders is one method for improving the required physicochemical and biological properties. Magnesium (Mg^2+^) is one of the four most abundant cations in the human body and plays a crucial role in bone metabolism and structural development [33,34,35]. Mg-containing materials have demonstrated enhanced biocompatibility, the ability to complete resorption in the body, and bioactivity concerning the formation of the bone matrix during osteogenesis [34]. This is due to their ability to have higher dissolution kinetics under in vivo conditions, which leads to faster bone resorption and remodelling due to the release of magnesium ions [36,37]. Mesenchymal stem cells (MSCs) are multipotent stromal cells normally found in bone marrow and blood, and they have the potential to form bones [7]. Recently, research showed that the introduction of Mg^2+^ ions into CPC can not only enhance cell adhesion and spread but also promote the osteogenic differentiation of MSC cells in vitro, enhance the formation of bones, and improve the growth of bones [38]. However, only a few studies have examined the regulatory effects of Mg^2+^ ions on MSCs in bone cements, biomedical materials, or magnesium ions, resulting in a lack of broad consensus.

Magnesium phosphate phases, along with calcium phosphate phases, have come to be regarded as the optimal material for bone defect replacement. Currently, considerable efforts are directed towards the study of bone cements based on magnesium phosphate minerals, such as struvite (MgNH_4_PO_4_·6H_2_O) or newberyite (MgHPO_4_·3H_2_O), which are promising alternatives to CPCs [39].

Improving the injectability and anti-washout ability of bone cements is an important task in adapting inorganic compositions based on calcium and magnesium phosphates for minimally invasive surgeries, including vertebroplasty and kyphoplasty. One of the most common approaches is linked with the introduction of polymer substances in cement compositions [40]. CPCs were mixed with gelatin, lactic-co-glycolic acid, poly (trimethylene carbonate) [41], lactide-co-glycolide [42], carboxymethyl cellulose [43], chitosan oligosaccharide [44], etc. [45,46,47,48]. Hyaluronic acid is a natural, non-toxic linear glycosaminoglycan that is widely used in pharmacological applications, particularly in ophthalmology, rheumatology, and dermatology [49]. This acid is present in many strains of bacteria and is ubiquitous in all vertebrates, where it is particularly abundant in the embryonic tissues and extracellular matrix of adult soft connective tissues [49]. It is characterised by hydrophilicity, non-immunogenicity, and good biocompatibility. The introduction of hyaluronic acid into CPC led to changes in cohesion [50] depending on the molecular weight and concentration. Moreover, it increased the proliferation and osteogenesis of osteoblasts [51], facilitating bone repair effects due to accelerated osteogenic expression [52]. Importantly, in 1986, Balazs proposed “hyaluronan” as an alternative to “hyaluronic acid”, as the carboxyl groups of the molecule are dissociated and thus attract cations, such as Na^+^, at physiological pH levels [53]. Sodium hyaluronate (hyaluronan, NaHA) was demonstrated as an efficient injection material for joint surgery [54], including osteoarthritis treatment [55,56] and persistent shoulder pain from various etiologies [57]. Then, when introduced in CPC, hyaluronan demonstrated an increase in wash-out resistance and the elongation of setting and injectable times [58]. At the same time, there is very limited data on the introduction of hyaluronic acid or NaHA into magnesium phosphate or magnesium–calcium phosphate cements (MCPCs). Previously, we have demonstrated improved injectability on the hydroxyapatite-contained MCPC, but biological properties were not evaluated [59]. It should be noted that N-acetyl and carboxylate groups in the hyaluronate of NaHA could interact with cations, including Ca^2+^ and Mg^2+^, and affect MCPC properties. This study aims to develop MCPCs with different concentrations of the Mg^2+^ cation to improve mechanical properties and degradation kinetics and to evaluate the effect on osteogenic differentiation. Moreover, the use of the biopolymer NaHA could improve the injectability of these cements. These polymer-based cements are sufficiently soft and can be moulded into bone defects and extruded from a syringe, which is important for the repair of complex-shaped bone defects. Thus, the aim of this study is to evaluate the physicochemical and biological behaviour of MCPCs based on the whitlockite and stanfieldite phases in the presence of NaHA.

## 2. Results

### 2.1. Characterisation of Cement Powders

The ICP data are presented in Table 1. According to the obtained results, the synthesised materials are characterised by a (Ca+Mg)/P ratio that is close to the predicted one.

The analysis of cement powders according to the laser diffraction data established that the powder particles did not exceed 100 µm in size. The decline in the characteristic values of D50 and D90 is indicative of a substantial decrease of lower than 100 µm in the coarse fraction content, which is important in terms of cohesion and injectability [60,61]. The absence of large particles also permits the uniform formation of cement phases. The cement powder with 20 mol.% of Mg^2+^ is characterised by smaller particles, despite the same grinding conditions. As magnesium content increased, the size of the coarse fraction increased from 18–20 µm to 40–50 µm.

According to X-ray data results, cement powders including 20 mol.% Mg^2+^ are characterised by the formation of magnesium-substituted whitlockite (Mg−Wt) Ca_2.589_Mg_0.411_(PO_4_)_2_, which exhibits trigonal syngony with parameters a = 10.345(4) Å and c = 37.263(9) Å in the amount of 84 wt.%. The main phase of the cement powders with 60 mol.% Mg^2+^ was stanfieldite (St)—Ca_3_Mg_3_(PO_4_)_4_—which exhibited monoclinic syngony with the following unit cell parameters: a = 22.809(2) Å, b = 9.996(1) Å, and c = 17.052(4) Å. Both powders are characterised by the formation of MgO as a minor phase in the amount of 5–10 wt.%.

**Figure 1:**
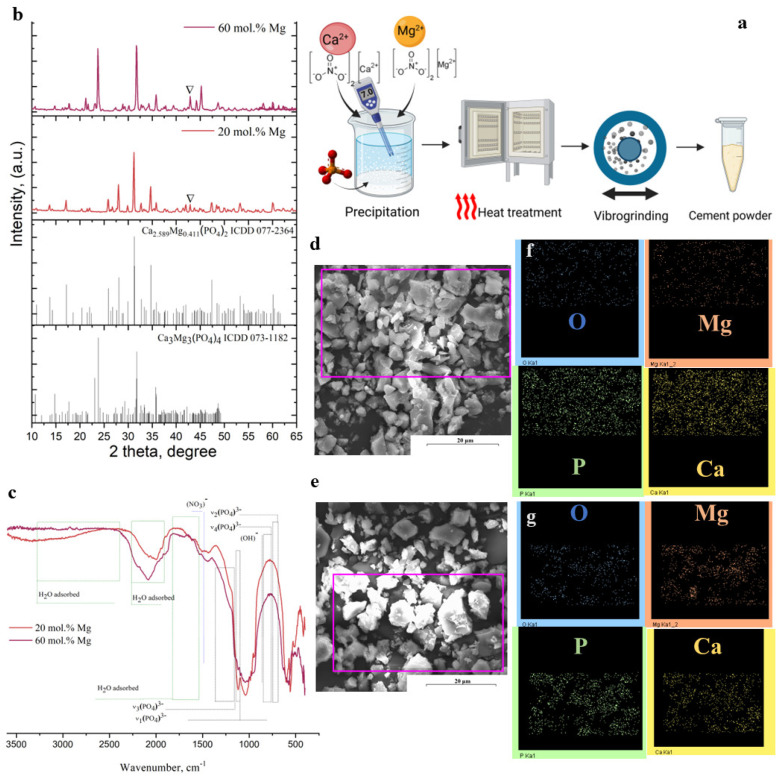
Scheme of the preparation of powders (**a**); phase composition according to XRD (**b**); FTIR spectra (**c**); powder morphology according to SEM; distribution of elements (Mg, Ca, O, and P) in 20 mol.% Mg (**d**) and 60 mol.% Mg (**e**) according to EDX in the mapping mode. The purple boxes in (**d**) and (**e**) are the area of element mapping, which presented on (**f**) and (**g**).

The SEM data of the 20 mol.% Mg^2+^ cement powder confirmed the formation of particles with a major fraction and a size of 20 μm, while 60 mol.% Mg^2+^ was characterised by particles of 50 μm. (Figure 1d,e). According to EDX data, this method detected the presence of Ca, Mg, P, and O in cement powders; the map of the cations’ distribution is presented in Figure 1f,g.

The FTIR spectra of the powders were characterised by a weakly resolved vibration region of carbonate groups (1570–1360 cm^−1^) [62] (Figure 1c). In all samples, there were no vibrations of hydroxyl groups (at 3570 cm^−1^), which could indicate the formation of hydroxyapatite [63]. Considering the main peak of phosphate vibrations in the region of 1300–800 cm^−1^, it should be noted that, for cement powder with 60 mol.% of Mg^2+^, there is an additional band at 1212 cm^−1^. Previously, Cacciotti et al. [64] related this band to the symmetrical stretching vibration of the P–O–P group in pyrophosphates.

### 2.2. Characterisation of Cement Liquids

The effect of the introduction of NaHA on cement liquid properties was determined at a concentration of 1.00 wt.%. High concentrations of NaHA negatively affect the cohesion of cements [50] and have limited solubility in saturated sodium phosphate solutions. For CPC cements, cement liquids based on an aqueous solution of NaHA were reported; NaHA was dissolved in a phosphate buffer [58], or hyaluronic acid was dissolved in citric acid [52].

The investigation of viscosity at different rotational speeds (Table 2) has shown that the introduction of polymers increases the viscosity of cement liquid by 6 times. The analysis of surface tension data shows that the introduction of NaHA into cement liquid has practically no effect on changes in this characteristic. The pH of the cement liquids was within the range of 3.4 ± 0.1 and was not affected by the introduction of NaHA.

### 2.3. Characterisation of Cement Materials

**Figure 2:**
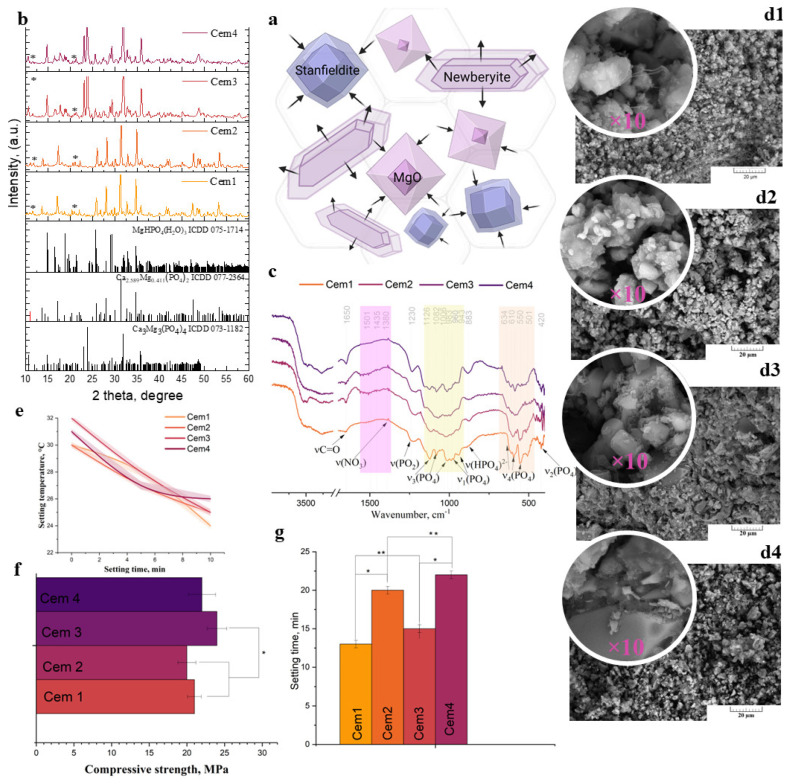
Scheme of cement phase formation (**a**); cement phase composition according to XRD, where * indicates the peaks of brushite phases; (**b**) cement samples’ FTIR spectra (**c**); temperature of the samples during hardening (**e**); compressive strength, where * denotes a significant difference between the cements, including differences in those containing Mg^2+^ (*p* < 0.05) (**f**); setting time, where * denotes a significant difference between the cements without and with NaHA (*p* < 0.05) and ** denotes a significant difference between the cements, including differences in those containing Mg^2+^ (*p* < 0.05) (**g**); and microstructure of the Cem1 (**d1**), Cem2 (**d2**), Cem3 (**d3**), and Cem4 (**d4**) samples.

Cement paste formation is linked to the partial dissolution of crystallinity phases in cement powder, including Mg-Wt and St, and the complete dissolution of magnesium oxide [60]. Cement formation is linked to the growth of new phases on the surface of the initial crystals, indicating an interaction between cement liquids and crystallinity phases, which plays a role as a Ca^2+^ and Mg^2+^ source according to schemes (1) and (2) (Figure 2b).

Ca_2.589_Mg_0.411_(PO_4_)_2_ + MgO + NaH_2_PO_4_·2H_2_O + H_2_O → CaHPO_4_·2H_2_O + MgHPO_4_·3H_2_O + Na_2_HPO_4_ (1)

Ca_3_Mg_3_(PO_4_)_4_ + MgO + NaH_2_PO_4_·2H_2_O + H_2_O → MgHPO_4_·3H_2_O + CaHPO_4_·2H_2_O + Na_2_HPO_4_ (2)

Cement materials based on cement powders were obtained and named Cem1–Cem4, including 20 mol.% Mg^2+^ and cement liquid without NaHA (Cem 1); cement powders including 20 mol.% Mg^2+^ and cement liquid with 1.00 wt.% NaHA polymers (Cem 2); and cement materials based on 60 mol.% Mg without NaHA and with NaHA (Cem 3 and Cem4, respectively). According to XRD (Figure 2a), after setting and hardening, the cements exhibited the presence of initial phases, namely, Mg-Wt or St, depending on the composition, and the formation of new cement phases was also observed: newberyite (MgHPO_4_·3H_2_O) and brushite (CaHPO_4_·2H_2_O). There were no magnesium oxide peaks in the materials after interactions with the cement liquid; the complete dissolution of magnesium oxide was observed. It should be noted that the introduction of the NaHA polymer into the cement liquid results in a decrease in the dissolution rate of cement materials, which is reflected in a decrease in intensity of newberyite peaks; moreover, the preservation of high-intensity St and Mg-Wtpeaks was observed in cement materials, as well as an increase in setting times.

The data obtained are confirmed via Fourier infrared spectroscopy studies (Figure 2c). It was observed that the spectra of all compositions contain broad and intense bands of adsorbed water at 3600–2500 cm^−1^ and 1600–1610 cm^−1^. The intensity of the valence vibration bands at 3163, 3267, and 3636 cm^−1^ for the OH group of three non-equivalent H_2_O molecules in newberyite decreases with the introduction of NaHA. Cem1 double peaks at 3545 and 3485 cm^−1^ and the peaks at 3286 cm^−1^ are characteristic of the OH stretching bands resulting from the hydrogen interaction of the OH group of cements with the OH group of NaHA. The band at 1230–1250 cm^−1^ corresponds to the antisymmetric stretching vibration of the bridging PO_2_ [65]. Simultaneously, the characteristic P-OH bands at 883 cm^−1^, related to HPO_4_^2−^, are present in the spectra. The decrease in intensity of the ν_1_ PO_4_^3−^ band at 960 cm^−1^ and the doublet of the ν_2_ PO_4_^3−^ mode at 510–520 cm^−1^ with magnesium concentrations increasing from 20 to 60 mol.% is associated with the transformation of the Mg-Wt structure into a St structure [66]. At the same time, the resolution of the ν_4_ PO_4_^3−^ bands at 634 cm^−1^ and 610–501 cm^−1^ decreases with the introduction of NaHA in the cement liquid. In the spectra of cements with NaHA, absorption bands at 1650 cm^−1^ also appear, which are related to the vibrations of the C=O polymer group [67].

According to SEM, the cement samples exhibited a loose and porous structure. The pore size range was from 1–2 to 6–8 μm. The enhancement of Mg^2+^ content resulted in a substantial increase in the density of the structure. The presence of NaHA is indicated by the local appearance of polymeric fibres (see first picture), which are visible at higher magnification. The surface of the cement was rough, with visible particles and micro-/macropores concentrated on the surface.

Understanding the functional properties of injectable bone cements is really important to quantitatively define setting processes, hardening mechanisms, and injection processes. To further improve cement properties such as cohesion, injectability, viscosity, and mechanical properties, the rheological properties of MCPC can be studied to gain insight into the intrinsic behaviour of the cement paste.

No exothermic reaction was observed during cement paste mixing. The startup temperature for Cem1 and Cem2 was ~30 ± 2 °C; then, the temperature decreased gradually to ~24 ± 2 °C (Figure 2e). Increasing the concentration of Mg^2+^ up to 60 mol.% in the cement powders indicated an increase in the startup temperature of ~32 ± 2 °C, followed by a decrease to ~26 ± 2 °C within 10 min of measurement (Figure 2e). It is known that magnesium phosphate cement MCPs are characterised by a rather strong exothermic reaction: up to 70 °C during the mixing of a cement powder with cement liquid [68]. The strong exothermic setting effect may damage the soft tissue around a bone and injure a portion of the bone marrow. The introduction of NaHA had no effect on temperature.

The obtained cement materials exhibited compressive strength within the range of 20–24 MPa, depending on the composition of powder and liquid (Figure 2f). The introduction of NaHA into the cements does not significantly reduce the strength of the cement materials. A significant difference was observed with respect to the increase in magnesium content in samples Cem1-2 to Cem3-4 due to the preservation of the St phase. Thus, the Cem3 samples demonstrated a compressive strength of up to 24.0 ± 2.0 MPa.

The setting time of cement materials without polymers is within the range of 10–12 min. With the introduction of NaHA, the setting time increased up to 20–22 min (Figure 2g). This effect is in contrast to the phenomenon previously described by Kai et al. [58]. This can be explained by the interaction of N-acetyl and carboxylate groups in hyaluronate (NaHA) with Ca^2+^ and Mg^2+^ ions from cement powders, along with the further preservation of excessive hyaluronate molecules, which bind hydroxy groups by hydrating and absorbing water molecules, resulting in setting time elongation. These data are confirmed via FTIR results. In the context of calcium phosphate brushite cements containing NaHA, it has been indicated that two factors influence the setting time [50]. The first factor is an increase in the viscosity of the medium, which impedes mass transfer and slows down the setting process. The second factor is the formation of complexes between the carboxyl groups of hyaluronate and calcium ions, which then serve as additional nucleation centres of the newly formed phases. In our case, calcium ions are not the sole participants in the complexation process; magnesium ions can also play a role. An increase in setting time was observed for brushite cements in the presence of low-molecular-weight sodium hyaluronate (300 kDa) in amounts of up to 0.5 wt.%. In this particular instance, despite the elevated content of sodium hyaluronate (100 kDa), a similar outcome is observed. The predominant factor is the increase in viscosity, which is manifested as an extension of the setting time from 10–12 min to 20–22 min.

**Figure 3:**
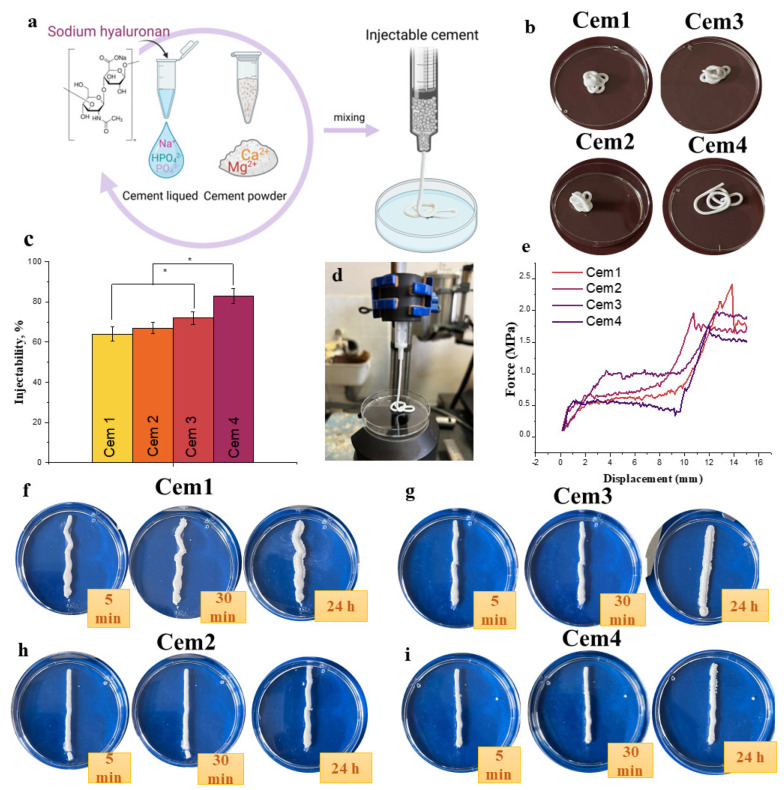
Scheme of the injectable cement formation and investigation (**a**); digital photos of the cement filament after the injection (**b**) and injectivity test (**d**); injectability of the cement samples, where * denotes a significant difference between the cements without and with NaHA (*p* < 0.05) (**c**); the influence of the composition on the injection force (**e**); digital photo of the cohesion procedure and the evolution of a filament in the SBF solution (**f**–**i**).

Injectability can be defined as the ability of a paste to extrude through the syringe (Figure 3a). During injection, the extruded paste has to retain its homogeneity (Figure 3b). This is a key element for the development of injectable bone cements. In the literature, there is no unified standard procedure for measuring injectability. Usually, it is identified by the force required for the complete extraction or quantification of extruded material during a certain period. The results of the injectability test of cement materials demonstrated that the introduction of NaHA increases the yield of the cement paste (Figure 3c). Cement materials without polymer additive Cem1 were characterised by an injectability level of 64 ± 3.5%, which is close to the value of cement materials without magnesium CPC [52], while the introduction of polymers into cement materials based on the powders—containing 20 mol.% Mg^2+^—slightly increases injectability. For cement materials based on powders that contained 60 mol.% Mg^2+^ (Cem3), the level of injectability was approximately 72 ± 3.1%. The introduction of NaHA increases injectability to 83 ± 3.8% in the Cem4 composition. For magnesium phosphate MCP-based cements, the introduction of sodium alginate (SA) results in increased injectability and improved cohesion of materials in PBS liquid, but the authors do not provide numerical indices of injectability [69]. Commercially available bone cements are normally either injected with a syringe or, in the case of a putty-like consistency, directly moulded into the defect by hand. For applications via a syringe, a high injectability and a maximum injection force of 200 N are required [45,46]. Ideally, injectability should be as close as possible to 100%, which means that the entire cement paste is extruded. The “filter pressing” phenomenon—that is, the separation of powder and liquid within the syringe—must be avoided [47]. At the same time, the paste should be sufficiently viscous such that it does not flow directly out of the syringe. According to the data from a force–displacement graph, it is possible to estimate the pressure that was applied when the cement materials were extruded (Figure 3e). Thus, the highest pressure of 2.4 N/mm^−2^ was observed for the Cem1 samples, which confirms the increased resistance of the material exiting the syringe. For the Cem4 material, the maximum applied force was 1.7 N/mm^−2^; it can be concluded that the introduction of NaHA into the cement liquid has a positive effect on the wettability of the cement powder, resulting in an increase in injectability and a decrease in the applied pressure. It is worth noting that the extrusion of cement materials occurred in a homogenous filament, and there was no separation of the paste into liquid and solid phases, which indicates the uniformity of the cement material (Figure 3b). The cohesion of investigated materials in the SBF solution was investigated (Figure 3f–i). It was observed that the introduction of NaHA into the cement liquid provides a significant improvement in the scouring resistance of cements. Cem4 based on the powder with 60 mol.% Mg^2+^ had the most visible effect (Figure 3i).

### 2.4. In Vitro Dissolution Assays

The degradation kinetic behaviour of the cement materials is determined by weight loss, pH, ion release concentration, Ca^2+^, Mg^2+^, and Na^+^, and the profile is plotted as a function of time. From the obtained data, it can be concluded that the solubility of the cement materials is strongly related to the initial Mg^2+^ content in the cement powders. Thus, Cem1 and Cem2 exhibited low mass loss at approximately 5.1 ± 1.1% on the 1st day of the experiment. As the soaking time of the materials in the SBF liquid was increased, there was an increase in mass up to the 21st day of observation, which is clearly related to the recrystallisation processes on the surface of the samples, as will be further shown after SEM and X-ray studies.

The solubility assay of the cement materials based on the powders with 60 mol.% Mg^2+^ content—Cem3 and Cem4—revealed that, on the 1st day of the experiment, the mass loss of the samples reached a range of ~15 ± 2.6% regardless of the type of cement liquid. In general, during the initial stage of the dissolution process—characterised by the removal of the unreacted cement liquid—the subsequent characteristics of the behaviour of the cement materials in the SBF model solutions were indicative of processes taking place at the surface of the material, where interactions between the dissolved ions proceeded. It is worth noting that the further dissolution of cement materials slows down due to the saturation of the surrounding solutions and the establishment of ionic equilibrium. In the conditions of a living organism, washing liquid streams are constantly renewed, and there is a leaching of material, which potentially accelerates the dissolution process. Further observations of up to 21 days show negligible changes in mass loss, remaining within the range of 12.9 ± 2% for Cem3 and 14.6 ± 1.5% for Cem4, associated with the simultaneous solubility and recrystallisation processes of the new phases on the sample’s surface.

**Figure 4:**
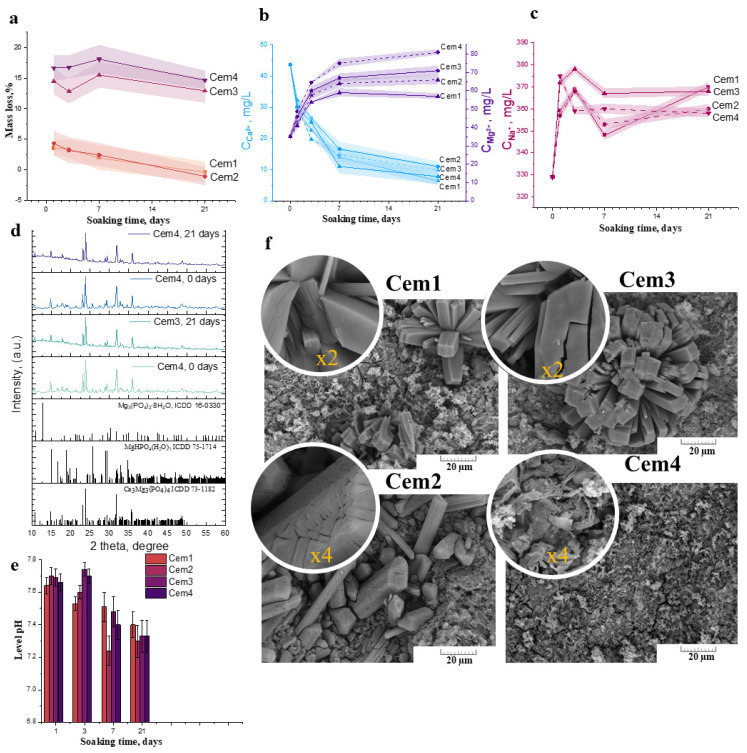
Mass loss (**a**); Ca^2+^ and Mg^2+^ release (**b**); Na^+^ release (**c**); XRD data (**d**); and morphology of the samples’ surface according to SEM during soaking (**f**); pH evolution in the SBF (**e**).

Secondly, ICP data showed that the dissolution of cement materials involved the active release of Mg^2+^ and Na^+^ cations. As soaking time increased, the concentration of these cations in the solutions rose, reaching a maximum at 21 days of the experiment. With respect to the dissolution of the cement-phase newberyite (solubility constant pKs° = 13.269 ± 0.113 at 37 °C), according to XRD data, complete dissolution occurs by day 7 of observation. Consequently, the most active release of magnesium occurs in magnesium-enriched materials. Thus, for sample Cem3, the concentration is equal to 71.2 ± 2.2 mg/L, and the introduction of NaHA results in an increase in magnesium concentration to 81.9 ± 1.2 mg/L. The same effect can be observed for materials based on 20 mol.% Mg^2+^-containing powders: the introduction of polymers increases the magnesium concentration to 66.5 ± 2.2 mg/L. The measurement of the pH value in the SBF solution of the cement materials at various time points exhibited neutral levels decreasing from 7.6–7.7 on the 1st day to 7.2–7.4 on the 21st day (Figure 4e).

According to the XRD results of soaking cement materials after 21 days, the newberyite phase decreased in quantity; in addition, the formation of magnesium phosphate bobierrite phase Mg_3_(PO_4_)_2_∙8H_2_O was observed (Figure 4d). The measurement of the pH value in the SBF solution of the cement materials at various time points exhibited a neutral level, which decreased from 7.6–7.7 on the 1st day to 7.2–7.4 on the 21st day (Figure 4e).

After 21 days of exposure, SEM data exhibited the formation of a porous and friable surface with flower-like structures (radial ray aggregates), indicating the newly formed bobierrite phase, which was confirmed as a magnesium phosphate phase via energy-dispersive X-ray analysis (EDX) (Figure 4f). The bobierrite phase was probably formed following reaction 6. The dissolution of newberyite may create a favourable basic environment for the synthesis of bobierrite according to reaction (3):

3Mg^2+^ + 2HPO_4_^2−^ + 8H_2_O → Mg_3_(PO_4_)2⋅8H_2_O + 2H^+^ (3)

### 2.5. In Vitro Cytotoxicity of MCPC Samples

**Figure 5:**
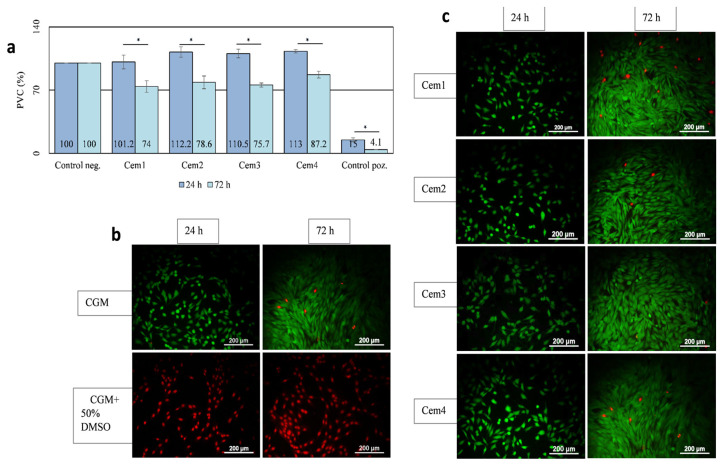
Viability of MG-63 cells after 24 and 72 h of growth in extracts of Cem1, Cem2, Cem3, and Cem4 cement samples (indirect test): (**a**) PVC of MG-63 in relation to control (polystyrene) and MTT test. Negative control—cells on polystyrene in CGM; positive control—cells in 50% DMSO. Error bars represent means ± SD for n = 3. *—Differences were identified as significant, which are represented by *p* ˂ 0.05. Border cytotoxicity value (70% of cell viability in CGM) is shown as a horizontal line; (**b**,**c**) fluorescence imaging of the live (green) and dead (red) staining of Mg-63 cells using the Live/Dead Kit.

The toxicity of Cem1, Cem2, Cem3, and Cem4 was assessed via an indirect test by studying the effect of their extracts on the viability of the MG-63 test culture at different cell growth periods. It was shown that after 24 h of cultivation, the population of viable cells on cement samples with 20 and 60 mol.% Mg^2+^ and two setting cement fluids did not differ or slightly exceeded the control values. After 72 h, the viability index in these groups decreased but remained above 70% (Figure 5a). The obtained results testify to the absence of the toxicity of the developed compositions of MCPCs, as evidenced by the results of experiments with MG-63 cells stained using a Live/Dead Kit (Figure 5b).

### 2.6. Effect of MCPC Samples on In Vitro Cytocompatibility

**Figure 6:**
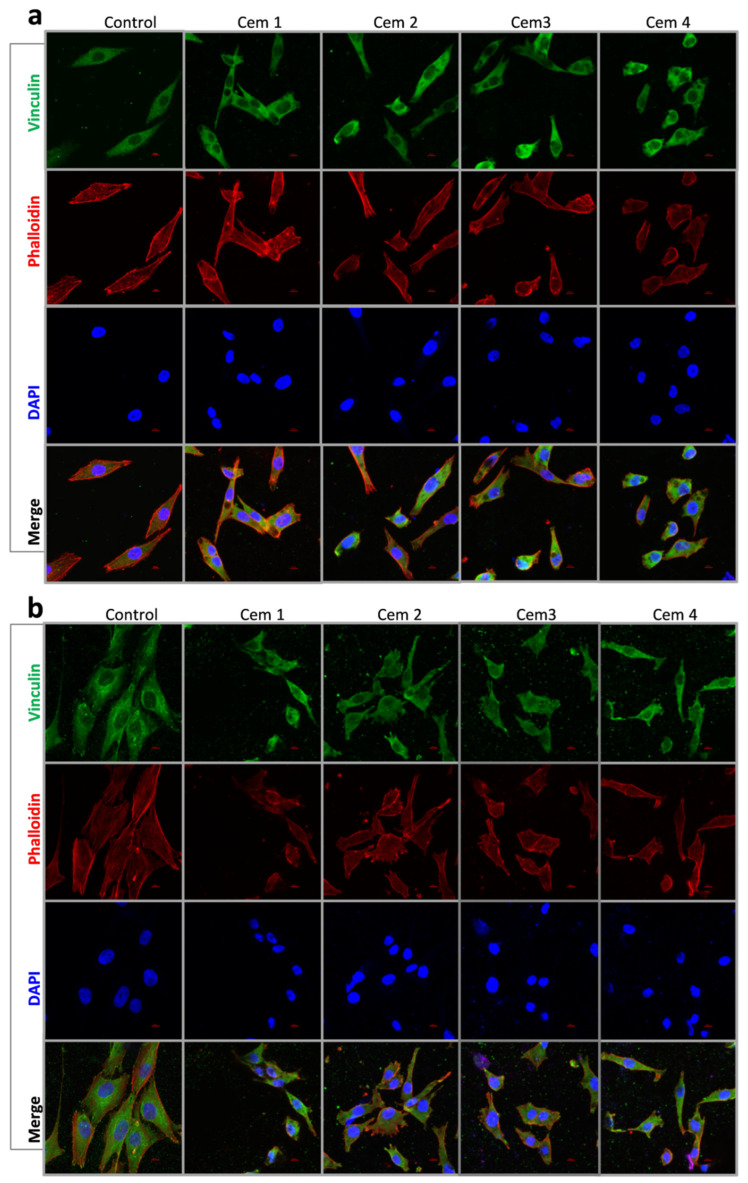
Immunofluorescence images of MG-63 cells in Cem1, Cem2, Cem3, and Cem4 stained with Vinculin (green), Falloidin (red), Dapi (blue), and Merge for 24 (**a**) and 72 (**b**) hours of cell growth, scale bar: 10 μm.

**Figure 7:**
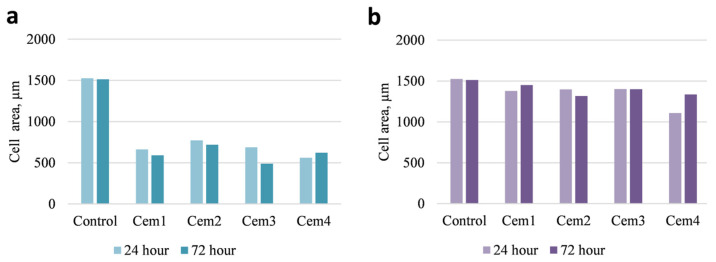
Area of MG-63 cells on cement samples (**a**) and on culture plastic positioned near the cement samples (**b**) after 24 and 72 h of cell growth.

**Figure 8:**
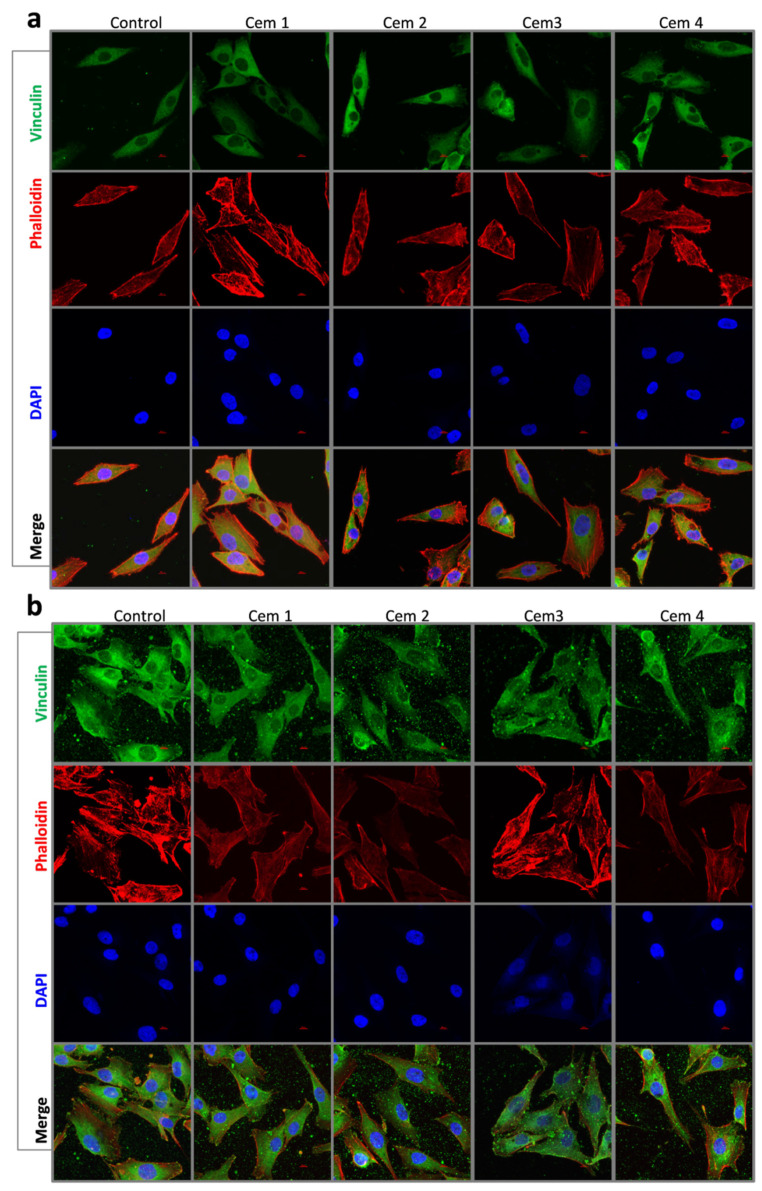
Immunofluorescence images of MG-63 cells on culture plastic near the Cem1, Cem2, Cem3, and Cem4 samples stained with Vinculin (green), Falloidin (red), Dapi (blue), and Merge for 24 (**a**) and 72 (**b**) hours of cell growth, scale bar: 10 μm.

To evaluate the influence of cement microstructure on the adhesion process of MG-63 cells, the morphology and area of cells were evaluated 24 and 72 h after seeding using fluorescence microscopy (Figure 6, Figure 7 and Figure 8). Additionally, the same parameters were also studied on a pool of cells attached to a culture plate next to the cement samples.

It was shown that 24 h after the beginning of the experiment, the MG-63 population on all cement samples was represented by smaller (compared to the control) cells of elongated and polygonal shape. The area of cells on cement samples was independent of the cement composition and ranged between 560 and 660 μm^2^ in the experiment vs. 1525 μm^2^ in the control (Figure 6a and Figure 7a). After 72 h of observation, the cells in the control and cement samples were mostly polygonal in shape and exhibited the same surface area (Figure 6b and Figure 7a).

Morphologically, a similar pattern was observed in the case of MG-63 cells attached to the culture plate next to the cement samples after 24 and 72 h of experimentation. The only difference was that the area of MG-63 cells was either slightly smaller or showed no statistically significant difference compared to the control (Figure 7b and Figure 8a,b).

**Figure 9:**
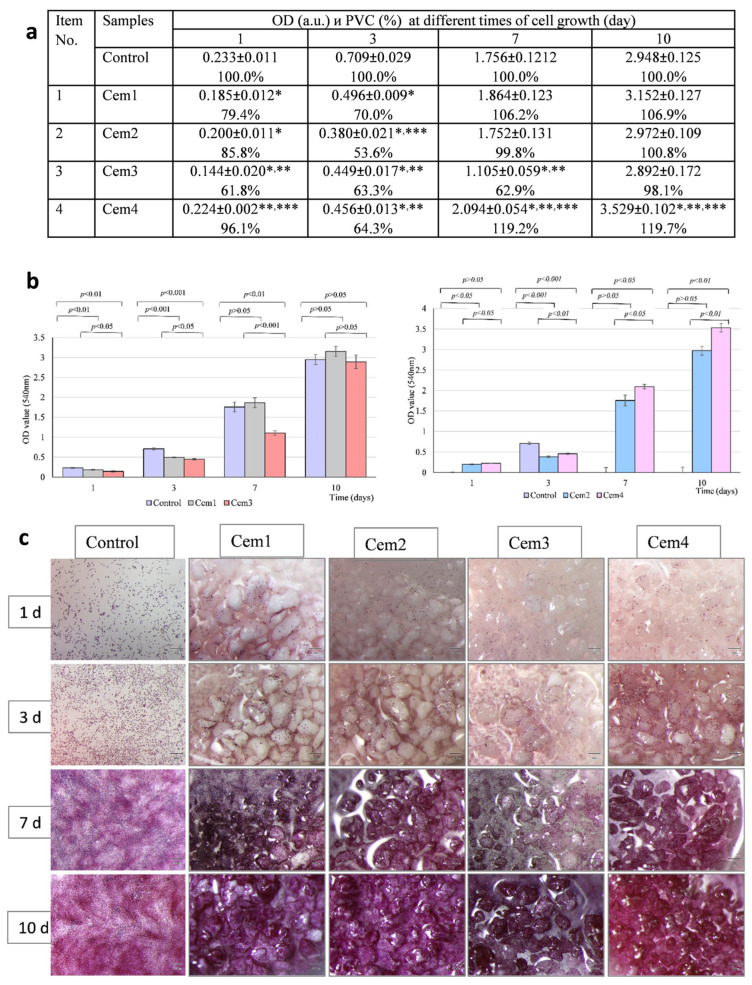
Cytocompatibility of Cem1, Cem2, Cem3, and Cem4 samples (direct test): (**a**) optical density of formazan solution (OD, MTT test, mean ± standard deviation, n = 3) and pool of viable cells (PVCs) of human osteosarcoma MG-63 cells on polystyrene (control) and experimental cement samples at different incubation times (significant difference determined via Student’s *t*-test (*p* ≤ 0.05): * between experimental groups and control; ** between experimental groups Cem 1 and Cem3, Cem2, and Cem4; *** significant difference between experimental groups Cem 1 and Cem2, Cem3, and Cem4. (**b**) Growth dynamics of human osteosarcoma MG-63 cells on polystyrene (control) and experimental bone cement samples Cem 1, Cem2, Cem3, and Cem4. (**c**) Expansion of the surface of bone cement samples Cem1, Cem2, Cem3, and Cem4 using MG-63 culture (1, 3, 7, and 10 days of cultivation, light microscopy x magnification, and MTT test). Scale bar: 300 μm.

To investigate the cytocompatibility of magnesium-containing bone cement samples, MG-63 cells were seeded directly onto experimental cement samples (direct test) and cultured for 10 days. According to the results of the MTT test, in the control (cells on polystyrene), the optical density value of the formazan solution, which indirectly indicates the size of the viable MG-63 cell population, increased from 0.233 units to 2.948 units from the 1st to the 10th day of cultivation. In the experiment, the optical density index of the formazan solution in the Cem1, Cem2, and Cem3 samples on day 1 and day 3 of cultivation—for all experimental cement samples—was lower than that of the control, which was statistically significant. However, after 7 and 10 days of observation, the proliferation rate of MG-63 cells on the surface of samples Cem1, Cem2, and Cem3 (exception—7th day for Cem3)—according to the optical density of the formazan solution—did not differ from the control, and for sample Cem4, it was higher than the control values, which was statistically significant. The effect of the amount of magnesium in the cement samples on the proliferative activity of the MG-63 cell test culture was analysed on the 10th day of cell expansion on the surface of the experimental bone cement samples. We found no statistically significant difference in the optical density value of the formazan solution for samples Cem1 and Cem3, whereas a significant difference in this parameter was found between samples Cem2 and Cem4: the population of Mg-63 cells on sample Cem4 was larger than the pool of MG-63 cells on sample Cem2 (Figure 9a,b), which was statistically significant.

Figure 9c shows the results of the efficiency of cell colonisation on the surface of the experimental MCPC samples in the dynamics of observation. It can be observed that in the early stages of the experiment (1st and 3rd day after sowing), the cells are located quite uniformly and sparsely in the control (polystyrene) and on all cement samples. An increase in the cultivation period was observed when there was an increase in their number. In the control, this resulted in the formation of a confluent monolayer at the bottom of the tablet; in the experiment, this led to the active expansion of MG-63 culture cells on the surface of cement granules and the formation of a single conglomerate of cement particles covered with cells on the 10th day in each experimental group.

In this part of the study, the cytocompatibility of experimental bone cement samples Cem1, Cem2, Cem3, and Cem4 was demonstrated via in vitro experiments.

### 2.7. Effect of MCPC on the Osteogenic Differentiation of BM hMSCs

To extend our analysis of the ability of the test cements to influence the osteogenic differentiation of BM hMSCs, we evaluated the expression of certain genes involved in the corresponding molecular mechanisms via RT-PCR. RT-PCR assays were performed on day 0 for untreated cells (undifferentiated control) and 2 weeks (day 14) after the addition of a cocktail of osteogenic factors to BM hMSCs cultured in the presence of Mg-substituted bone cements.

The expression of specific genes was determined and normalised to *GAPDH.* Fold-change (FC) values were calculated relative to day 14 MSCs growing in CGM. Among the genes important for osteogenesis, we assessed the expression levels of *RUNX2* (encoding runt-related transcription factor 2), *ALPL* (encoding tissue non-specific alkaline phosphatase), and *SP7* (osterix (*OSX*) transcription factor).

**Figure 10:**
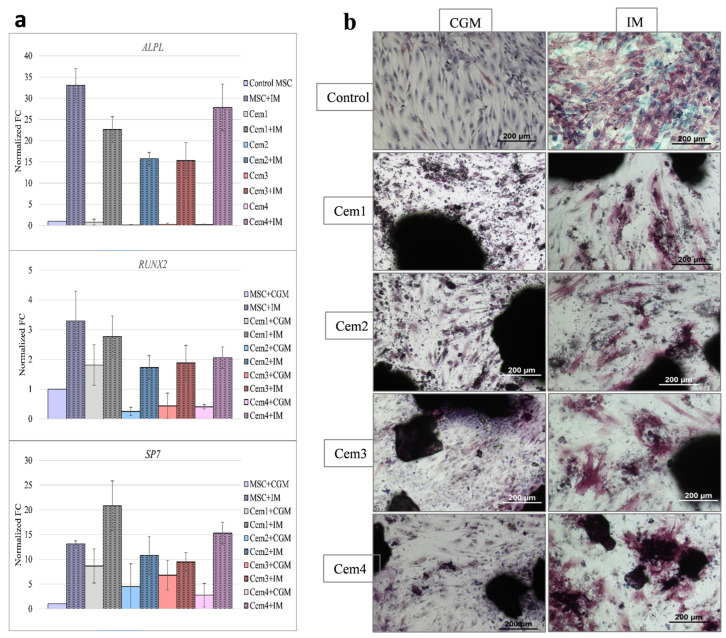
Effect of Cem1, Cem2, Cem3, and Cem 4 on BM MSC in vitro osteogenesis: (**a**) the expression levels of osteogenesis-related genes *ALPL, RUNX2*, and *SP7*. Error bars represent means ± SD for n = 3. Differences were identified as significant according to *p* < 0.05; (**b**) ALPL staining.

The addition of osteogenic differentiation factors significantly stimulated the expression of the *ALPL* gene in all MSC cultures regardless of the presence of bone cement (Figure 10). The change in the expression level of Cem4 was slightly higher compared to other compositions, but it did not reach statistical significance compared to the control MSC+OD culture. Simultaneously, the relative expression levels of *RUNX2* were not significantly different in the presence of all cement compositions. The expression of the *SP7* gene was significantly increased (*p* = 0.034) in the presence of Cem1 compared to MSCs cultured on pure plastic; however, a comparable level was also observed when Cem4 was added to the culture (Figure 10a).

In addition to the PCR analysis of the expression of several genes responsible for the osteogenic differentiation of donor BM MSCs, we also detected alkaline phosphatase in donor BM MSCs after culturing in special osteogenic medium via the immunocytochemical method (Figure 10b). It was shown that after 14 days of cell growth in a special osteogenic medium in the control and experimental groups, a portion of MSCs lost their typical spindle shape and acquired the appearance of widely spread cells. On slides, these cells were localised via purple staining. The most intense staining for alkaline phosphatase was detected in the preparations of sample Cem4.

These RT-PCR results are consistent with the cytological ALPL staining results described above when comparing the composition of Mg-substituted bone cements in terms of their ability to stimulate BM-MSC differentiation into the osteogenic lineage. Taken together, the results indicate that all tested bone cements can maintain osteoblast differentiation at the expression level of specific genes.

## 3. Discussion

Bone cements are mainly used for bone replacement in minimally invasive surgical procedures, and an injectable composition is therefore an attractive option. The main advantage of bone cements is their ability to fill any bone defect shape—they can be moulded to the shape of the bone and harden when injected in situ, which can reduce surgical time and postoperative pain. Research on the creation of new MCPCs for bone replacement and regeneration is urgently needed for modern materials science. Due to the unique composition of cement powders, they have more advantages compared to traditional CPC. Firstly, bone cements based on the calcium phosphate–magnesium phosphate system have previously exhibited higher biocompatibility with bone tissue, enabling better integration with the surrounding tissue and promoting faster regeneration [70,71]. Second, magnesium-containing bone cements exhibit higher mechanical properties, rendering them more reliable [37,72,73]. Thirdly, the transition to modern minimally invasive surgery to treat bone defects with injectable bone cements reduces the risk of complications, which in turn shortens rehabilitation time and improves the patient’s quality of life [74,75]. To meet these challenges and improve injectability, we developed NaHA-containing MCPCs. NaHA is a naturally occurring polysaccharide in organisms, and it is composed of alternately linked disaccharide units of *N*-acetylglucosamine and glucuronic acid, which are commonly found in living organisms. The molecular structure of HA contains a large number of hydroxyl (-OH) and carboxyl (-COOH) groups; thus, HA is highly absorbent and has strong moisturising properties. HA and NaHA are used to modify inorganic and organic compositions for bone tissue regeneration [76], including the creation of rigid scaffolds and colloids [77].

Cement powders containing different amounts of magnesium were obtained via the precipitation method. It was found that cement powders containing 20 mol.% Mg^2+^ are formed in the main phase of the trigonal configuration of Mg-Wt. When magnesium contents increased to 60 mol.% Mg^2+^, the monoclinic phase of St appeared. As is known from the literature, magnesium stabilises the whitlockite lattice [78], but when concentrations above 50 mol.% are reached, the St phase is crystallised. A cement powder containing about 10 mol.% MgO is obtained, which results in the formation of new cement phases during interactions with acidic cement liquid based on the contained NaH_2_PO_4_ and NaHA.

We developed cement compositions that demonstrated sufficient mechanical properties, which were comparable to the NaHA-containing CPC (up to 22 MPa in our study compared to the maximum value of 18.78 ± 1.83 MPa reported in [58]); acceptable setting times, injectability, and wash-out resistance were also observed. According to XRD and FTIR data, materials characterised by the formation of newberyite and brushite phases were developed, and they provide neutral pH levels and prevent temperature increases, similarly to our previous studies [66,75]. The introduction of NaHA resulted in an increase in setting time of up to 22 min, but this period is still appropriate [25] and significantly shorter than 55.3 ± 4.0 min in the case of NaHA-CPC [58], and it is close to 23.64 ± 0.76 min for hyaluronic-acid-containing CPC [52]. The molecular weight influence on the setting time was demonstrated, and low-molecular-weight hyaluronic acid—300 kDa introduction—increased the brushite cement setting time from 17 to 23 min, while high-molecular-weight hyaluronic acid—1649 kDa introduction—decreased the setting time to 13 min for CPC [50]. It is important to note that in the case of CPC, the introduction of NaHA resulted in a change in setting times due to the initial NaHA network’s formation during interactions with Ca^2+^. In contrast, in the case of MCPCs, NaHA resulted in the deceleration of newberyite crystallisation processes according to XRD, extending the state of the deformable cement paste. In this study, the addition of NaHA slightly increased the setting time and improved the injectability up to 83 ± 3.8%, possibly because water-soluble HA increased the viscosity of the cement liquid, supporting the appropriate level of mechanical strength—approximately 22.0 ± 1.8 MPa.

Cement performance influences from the point of view of cohesion properties are very important [79]. Previously, the CPC cement pastes used for vertebroplasty were found to induce blood clotting upon contact with blood, which was triggered by interfacial reactions between the blood and solid particles released from the CPC [80]. The introduction of viscous solutions, along with a decrease in particle size via grinding, is one of the most efficient strategies for improving cohesion resistance. In our study, we demonstrated that the introduction of NaHA significantly improved the cohesion of Cem2 compared to Cem1 compositions. The filament of Cem2 is characterised by the preservation of the filament’s shape during the 30 min point; after 24 h, the soaked filament starts to wash out. The high cohesion resistance was evaluated during 5, 30 min, and 24 h of observation for Cem3 and Cem4. It was also observed with NaHA’s introduction. We linked this effect to the higher newberyite amounts in the Cem3 and Cem4 compositions, which were more stable compared to brushite when placed in liquid. Compared with CPC-NaHA-modified bone cements, we observed improved shape preservation compared to samples with a low NaHA concentration, and similar results were also observed when 0.8–1.0 wt.% NaHA was introduced [58].

The average injectability of the cements significantly increased when NaHA was introduced into the composition. These results are in good agreement with previous observations on NaHA and hyaluronic-acid-containing CPC.

The degradation behaviour of the SBF solution demonstrated the strong influence of magnesium contents on the cement’s solubility. The mass loss of Cem1 and Cem2 was lower compared to Cem3 and Cem4. The newberyite phase was completely dissolved, and the bobierrite phase was detected on the 21st day of the experiment. The introduction of NaHA led to a further increase in mass loss, and magnesium was correspondingly released into the SBF solution.

The inclusion of magnesium ions in MCPCs to improve the bioactivity of developed bone cement formulations is a justified strategy, as this element is the fourth most abundant in the human body and fulfils many functions in maintaining the homeostasis and physiology of bone tissue. Magnesium is known to stimulate osteogenesis in vitro as part of alloys [81], coatings on titanium alloys [82], PLGA-based composite biomaterials [83], calcium phosphate cements [84], and bioceramics [85]. Importantly, we have uncovered the cellular and molecular mechanisms associated with the activation of key signalling pathways that mediate the effects of osteogenic differentiation in vitro.

In addition, the angiogenic and antibacterial effects of magnesium oxide in HA-based bioceramics were observed. Moreover, a therapeutic window of magnesium content was identified, at which the bioceramic remained cytocompatible, preserved the proliferative potential of osteoblasts, stimulated angiogenesis, and exhibited antibacterial activity [86].

Our in vitro biological studies on the obtained MCPC Cem1, Cem2, Cem3, and Cem4 allowed the conclusion of the absence of toxicity with respect to the developed cement compositions: the value of the fraction of surviving cells of the test culture of MG-63 cells after 24 and 72 h of observation exceeded 70%. The direct contact of the MG-63 culture with the surface of the developed bone cement compositions over a 10-day cultivation period confirmed their cytocompatibility, demonstrating the ability to support cell adhesion and active proliferation, which was most pronounced for the Cem4 composition.

Finally, in assessing the ability of these MCPCs to mediate the osteogenic differentiation of the BM hMSC culture when cultured in a specific induction medium, the expression of key osteogenesis genes *ALP, RUNX2*, and *SP7* was examined via RT PCR, and alkaline phosphatase levels were determined using the immunocytochemical qualitative method. It was observed that the *ALPL* gene was most actively expressed in the BM hMSC culture on the Cem4 sample, and alkaline phosphatase depositions were also observed, which were confirmed immunocytochemically. Alkaline phosphatase enzyme activity is an early marker of MSC differentiation into osteoblasts and the formation of osteoid, a non-mineralised bone matrix. The obtained results indicate that the developed compositions of MCPCs, together with cytocompatibility, support the morphogenetic potencies of MSCs, i.e., they are bioactive.

Our results strongly support the potential for further in vivo studies of the developed MCPC compositions for applications in reconstructive plastic surgery in onco-orthopaedics, maxillofacial surgery, and traumatology.

## 4. Materials and Methods

### 4.1. Chemicals

High-purity-grade calcium nitrate tetrahydrate (≥99.0), magnesium nitrate hexahydrate (≥99.0), diammonium phosphate (≥99.0), and sodium acid phosphate (≥99.0) were acquired from Labtech (Russia). Sodium hyaluronate with a molecular weight of 100 kDa was from Sigma Aldrich, St. Louis, MO, USA (≥99.0).

### 4.2. Preparation of Cement Powders, Cement Liquid, and Samples

(10 − 10x)Ca(NO_3_)_2_ + 10xMg(NO_3_)_2_ + 8NH_4_OH + 6(NH_4_)_2_HPO_4_→

Ca_(10*−*10x)_Mg_10x_(PO_4_)_6_(OH)_2_ + 20NH_4_NO_3_ + 6H_2_O, (4)

Here, x = 0.02; 0.06.

The powders were obtained via precipitation, as previously described by the authors of [66]. Briefly, calcium nitrate and magnesium nitrate solutions were prepared and dropwise added to diammonium phosphate ((NH_4_)_2_HPO_4_) with constant stirring, and a pH of approximately 7.0 ± 0.1 was maintained using an aqueous ammonia solution (NH_4_OH). After precipitation, to preserve the predicted chemical composition, the liquid phase was evaporated from the precipitated powders. The precipitates were subjected to heat treatment at 300 °C for 6 h; they were then calcined in air for 2 h at 1150 °C for materials containing 60 mol.% Mg^2+^ and at 1350 °C for those containing 20 mol.% Mg^2+^. The sintered blocks were milled in an agate mortar and then ground in a planetary ball mill, which was equipped with Teflon vessels with zirconia balls, for 80 min in the air. According to the CaO–P_2_O_5_–MgO phase diagram, all major phases in the obtained cement powders were crystallised after heat treatment.

A solution based on 100 wt.% NaH_2_PO_4_, characterised by a pH value of 3.4 ± 0.1, was prepared using an overhead stirrer containing salt in deionised water, and the solution was served as cement liquid. In order to improve the injectability of cement materials, sodium hyaluronate (NaHA) was introduced into the cement liquid in amounts of 1.00 wt.%. Cement materials were prepared by mixing a cement powder with the cement liquid at a powder-to-liquid ratio of PLR = 2; this was carried out under sterile conditions on a glass slide using a spatula. For the characterisation of materials and for the mechanical tests, cement samples were prepared in cylindrical form with a diameter of 0.8 mm. After setting, the samples were hardened in an atmosphere with 100% humidity for 24 h.

Cement materials were obtained and labelled as follows: Cem1, Cem2, Cem3, and Cem4.

### 4.3. Characterisation of the Materials

The chemical composition of the powders was analysed via inductively coupled plasma atomic emission spectrometry (AES-ICP, Agilent 720, Vista Pro, ICP Expert 4.0 Software). The uncertainty of the ICP results did not exceed 2.5%.

The granulometric composition of cement powders was examined using a FRITSCH Analysis 22 laser particle analyser. The phase composition of the materials was determined via X-ray phase analysis (XRD) (on a Shimadzu XRD-6000, Kyoto, Japan) using CuK_α_ radiation within the 2θ range from 10° to 60° with a step of 0.02° using the ICDD database PDF2. The phases were matched with the following cards: stanfieldite (Mg_3_Ca_3_(PO_4_)_4_: ICDD 000-73-1182); newberyite (MgHPO_4_·3H_2_O: ICDD 969-00-7633); magnesium oxide (MgO: ICDD 000-77-2364); brushite (CaHPO_4_∙2H_2_O: ICDD 000-11-0293); magnesium phosphate (Mg_3_(PO_4_)_2_: ICDD 000-071-1164); whitlockite (Ca_3_(PO_4_)_2_: ICDD 000-09-0169); and bobierrite (Mg_3_(PO_4_)_2_∙8H_2_O: ICDD 000-16-0330). For the evaluation of whitlockite-type phases, magnesium-substituted whitlockite phases ((Ca_2.589_Mg_0.411_ (PO_4_)_2_: ICDD 000-87-1582) and (Ca_18_Mg_2_H_2_(PO_4_)_14_: ICDD 000-70-2064)) were also considered. The phase composition was determined using the PHAN% software. The uncertainty of the XRD results was 5%.

The cement powder’s morphology and the microstructure of set cement materials were investigated via scanning electron microscopy (SEM) (Tescan Vega II, Brno, Czech Republic). Energy-dispersive X-ray analysis was performed in the mapping mode using SEM to establish chemical compositions with the help of an Oxford Instruments detector (Oxford, UK). An analysis of the SEM data was carried out using ImageJ software 1.52u with the secant method applied to at least 50 line segments.

The Fourier transform infrared (FTIR) spectra of the samples were obtained via the KBr technique using a Nicolet Avatar-330 FTIR spectrometer (ThermoFisher Scientific, Waltham, MA, USA) in the range from 4000 to 400 cm^−1^ to evaluate the functional groups of the samples.

The setting time of cement materials was determined by plunging a 1.0 mm (400 g) Vick needle with an indenter diameter of 1.0 mm (400 g) into the sample until the needle no longer forms a complete circular depression in the cement sample, in accordance with the authors of [87]. The pH values of the extracts were determined in distilled water using Testo pH1 equipment.

The viscosity of the cement fluid solutions was measured on a Brookfield DV2T viscometer (MSA Altair) at 25 and 100 rpm.

### 4.4. Mechanical Testing

An Instron 5581 uniaxial testing machine was employed to measure the compressive strength of the cement samples at a crosshead speed of 1 mm/min according to ASTM D695-91. Five samples of each composition were analysed to determine the compressive strength, and the results are reported as mean ± standard deviation (SD).

### 4.5. Injectability and Washout Resistance

The injectability of the cement paste was measured in vitro using a method described previously [52]. Injectability was assessed by manually pushing paste-like cement through a 2 mL syringe with an inner diameter of 10 mm, wall thickness of 0.9 mm, and an extrusion aperture inner diameter of 3 mm. The filled syringes were fixed perpendicularly in a customised matrix and positioned between the knock plates of a compression machine. The injectability of cement samples was investigated by measuring the mass of a material extruded through a syringe at constant pressure, in accordance with the authors of [87]. This method was optimised, and the analyses were carried out using an Instron 5581 instrument.

The methodology of the experiment consisted of several stages. At the first stage, the mixing of a cement powder with the cement liquid at PLRs = 2 was performed on a glass slide with a spatula. The mixtures were blended manually for 3 min. Then, the cement paste was transferred into a syringe. The volume of the cement material in the syringe was 1 mL. During the second stage, the syringe with the cement paste was immersed in the die of the unit, and pressure was applied during a constant transverse movement of 1 mm/s to the punch of the syringe, with a maximum travel distance of 15 mm (velocity measurement error: 0.2%; load measurement error: 0.5%) for all samples.

Injectability was calculated using the following equation:

(5) $$\mathrm{Injectability}\;(\%)\;=\;\frac{W_{1}-W_{2}}{W_{1}}\times 100\%$$

where w_1_ is the initial mass of 1 mL of the material in the syringe before the experiment, and w_2_ is the mass of the extruded material.

Cohesion was evaluated by visually inspecting the sample via a modified method. For the anti-washout ability test, the prepared pastes were loaded into a 2 mL syringe and then directly injected into the SBF solution. The samples were observed for disintegration in the solution before and after they were combined with the SBF solution at 37 °C for 5 and 30 min and 24 h via gentle shaking. The disintegration of the composites was examined according to the authors of [63].

### 4.6. Dissolution Assays

In vitro passive degradation assays were performed by evaluating weight loss, phase composition changes, and microstructure. To understand the degradation behaviour and solubility of MCPCs, samples in the form of discs (6 mm in diameter and 2 mm in height) were soaked in SBF at 37.0 °C, and a *w*/*v* ratio of 0.2 g/mL and a surface area-to-volume ratio of 0.1 cm^−1^ were used for various periods in a closed system. Two samples were taken from the SBF at each predetermined time point (1, 3, 7, and 21 days), and they were then washed gently with distilled water and lyophilised for 24 h. Surface morphologies and compositions were determined via SEM and XRD. The ion release into the solution (i.e., into SBF) and the concentration of Ca, Mg, and Na ions in the solutions after soaking for different periods were determined via ICP-MS measurements.

### 4.7. In Vitro Assay

Prior to in vitro studies, MCPC samples were sterilised via γ-irradiation at a dose of 18 kGy.

#### 4.7.1. Cell Cultures

The MG-63 human osteosarcoma cell line (Russian Cell Culture Collection, Institute of Cytology, Russian Academy of Sciences, St. Petersburg, Russia) was used as a test culture. The culture was maintained at an initial seeding density of 10.0 × 10^3^ cells/cm^2^ in a complete growth medium (CGM) with the following composition: DMEM medium (PanEco, Moscow, Russia), 10% foetal calf serum (FBS, HyClone, Logan, Utah, USA), 0.58 mg/mL of L-glutamine (PanEco, Moscow, Russia), 20.0 µM of Hepes solution (PanEco, Moscow, Russia), and gentamicin solution (50 µg/mL, Dalchimpharm, Moscow, Russia).

To assess the osteodifferentiation potential of MCPC samples, experiments were performed on the culture of donor bone marrow mesenchymal stromal cells (BM hMSCs) of the 4th passage, which was maintained at a seeding density of 1.0 × 10^3^ cells/cm^2^ in the CGM of similar (as for MG-63) compositions, except for the culture medium DMEM/F12 (PanEco, Russia) (informed consent was provided by the donor and approved during a meeting with the Local Ethical Committee of P. A. Herzen MNIOI—branch of FGBU “NMRC Radiology” of the Ministry of Health of the Russian Federation; protocol No. 637 dated 15 February 2021).

To verify the “stemness” of the BM hMSC culture, the immunophenotyping of this culture was performed using a specific set of monoclonal antibodies (MABs) against differentiation clusters: CD105, CD90, CD73, CD45, and CD34 (Miltenyi Biotec, Gladbach, Germany, flow cytometry Cytoflex Beckman Coulter, Inc. Miami, Fl, USA). Moreover, the ability to induce differentiation in the osteogenic direction was determined by culturing BM hMSCs in a specific differentiation medium (StemPro Osteogenesis Differentiation Kit, Stemcell Technologies, Vancouver, BC, Canada). At the end of the culture period, the marker of osteogenic differentiation—alkaline phosphatase (alkaline phosphatase staining kit, Himedia, Mumbai, India)—was determined in the cells. BM hMSCs with CD90+, CD105+, CD73+, CD34-, and CD45- phenotypes, capable of osteogenic differentiation when cultured in a specific induction medium, were used.

The passage of MG-63 cells and BM hMSCs was performed according to a standard technique, with medium changes carried out twice a week. When the culture reached 80% confluence, the cells were suspended with a mixture of 0.25% trypsin (PanEco, Russia) and Versene (PanEco, Moscow, Russia) at a ratio of 1:1, counted on a Countess II FL automated cell counter (Invitrogen, ThermoFisher Scientific, Life Technologies, Carlsbad, Ca, USA), and dispersed in culture vessels (Corning Inc., Corning, NY, USA) at a ratio of 1:3.

All manipulations with materials and cells were carried out under sterile box conditions in a laminar flow cabinet of protection class II (Heraeus, HERAsafe, Hanau, Germany). Cell plates were cultured in a humid atmosphere of a CO_2_ incubator (Sanyo MCO-20AIC, Sanyo Electric Co., Osaca, Japan) at 37 °C and 5% CO_2_.

#### 4.7.2. Cell Viability Determination Methods

##### MTT Test

The method is based on the ability of living cell dehydrogenases to reduce 3-(-4,5-dimethylthiazolyl-2)-2,5-diphenyltetrazolium bromide (MTT, Sigma Aldrich solutions, Merck SA, Darmstadt, Germany) to blue, water-insoluble formazan crystals. It is known that the amount of formazan formed can characterise not only the metabolic but also the proliferative activity (viability/quantity) of different types of human and animal cells. For the MTT test, at the end of the cultivation, 50 µL of ORS was taken from each well, and 25 µL of the MTT solution at a concentration of 5 mg/mL was added if the experiment was performed in 96-well plates. Moreover, 250 µL was taken, and 125 µL of MTT was added if 48-well plates were used. After incubation for 3 h (5% CO_2_, 37 °C), the entire volume of medium was decanted from each well. The resulting formazan was dissolved with isopropyl alcohol (200 µL per well in a 96-well plate or 1000 µL in a 48-well plate) (Himmed, Moscow, Russia). The precipitate resulting from protein precipitation in isopropanol was removed via the centrifugation of plates for 10 min at 3000 rpm (Jouan BR 3.11, ThermoFisher Scientific, Life Technologies, Carlsbad, Ca, USA). Then, 100 µL of the supernatant from each well was transferred to a 96-well flat-bottomed plate (Costar, EIA/RIA, Corning Inc., Corning, NY, USA), and the optical density (OD) of the formazan solution was estimated on a Multiscan FC spectrophotometer (ThermoFisher Scientific, Life Technologies, Carlsbad, CA, USA) at a wavelength of 540 nm.

A sample of MCPC was considered non-toxic if IT≤30%, and it was considered cytocompatible if the PVC value was ≥70%.

At the experimental stages, we also carried out the visual control of cell expansion processes on the sample surface and photographic archiving of materials (Olympus stereomicroscope systrm SZX7, Olympus, Tokyo, Japan).

##### Live/Dead Assay

The determination of cell viability using the Live/Dead Kit (Molecular Probes Invitrogen ThermoFisher Scientific, Life Technologies, Carlsbad, CA, USA) was performed according to the manufacturer’s protocol. For the visualisation of live and dead cells, the MG-63 culture came into indirect contact with the surface of the samples under investigation for the simultaneous fluorescent staining of live (using calcein-acetoxymethyl (calcein-AM)) and dead (using ethidium homodimer (Eth-1)) cells. Calcein, formed via the cleavage of calcein-AM by esterases in living cells, fluoresces green at λ = 517 nm; the nuclear dye Eth-1 only enters the nucleus through the ruptured membranes of dead cells and fluoresces red at λ = 617 nm.

We then carried out microscopy on the stained cultures in the experiment and the control groups (Nikon Eclipse TI fluorescence microscope, Tokyo, Japan).

#### 4.7.3. Assessment of Cytotoxicity of MCPC Samples via the Indirect Contact Method

The acute toxicity of the tested samples was determined via the indirect contact method, in accordance with the authors of [88], by culturing test cultures in the extracts of the MCPC samples.

The extracts were prepared under sterile conditions in a thermostat at 37 °C for 24 h on a rotary shaker (ELMI, Riga, Latvia) and at a rotation speed of 170 rpm. CGM for cell cultures was used as the extraction fluid. According to the authors of [88], the material mass/volume ratio of the extraction liquid was 0.2 g/mL ± 10%.

The material particles trapped in the extracts during extraction were removed via centrifugation at 3000 rpm for 15 min (Eppendorf Centrifuge 5810 R, Hamburg, Germany). The extracts were used immediately after preparation.

The seeding density of the test culture—human osteosarcoma MG-63 cells—was 7.0 × 10^3^ cells per well (20.0 × 10^3^ cells per cm^2^, 96-well culture plates, Corning Inc., Corning, NY, USA) in the 200 μL CGM volume. Sample extracts were added to the cells 24 h after seeding, when the MG-63 culture had formed a subconfluent monolayer. For this purpose, the culture medium was decanted completely from the wells, and extracts were added at 200 µL per well. CGM was added to the cells as a negative control, and a 50% solution of dimethyl sulfoxide (DMSO, PanEco, Moscow, Russia) was added to CGM as a positive control. At least 5 replicates were used for extracts from each material sample and control. The cell growth periods of the test cultures treated with MCPC sample extracts were 24 and 72 h. Cell viability was estimated using the MTT assay and Live/Dead Kit.

For each MCPC sample under study, in a specific cell growth period, we calculated the population of viable cells (PVCs) in the experiment relative to the control (in %) using the following formula:

PVC = OD-experience: OD-control × 100%,

where OD denotes the optical density value of the formazan solution in the experiment and in the negative control. Furthermore, in assessing the toxicity of the samples, the toxicity index (IT) was calculated using the following formula:

IT = 100%—OD-experiment: OD-control × 100%

The material sample was considered non-toxic at IT ≤ 30%.

Cell expansion processes on the surface of samples were visually monitored using a stereomicroskopeOlympusJapan) and a Nikon Eclipse Ti microscope (Japan).

#### 4.7.4. Investigation of Adhesion Properties of the Surface of MCPC Samples

To evaluate the degree of cell adhesion to the surface of MCPC samples, the cell area was determined after 24 and 72 h of culturing. For this purpose, cement samples were placed at the bottom of 8-well slide culture bottles (Corning Inc., Corning, NY, USA), with each containing 1 type for each incubation period. Then, a suspension of MG-63 cells at a concentration of 10 × 10^3^ cells/cm^2^ was added to the wells with (experiment) and without samples (control, polystyrene). The culturing periods were 24 and 72 h.

At the indicated times, the cells were fixed with a 4% paraformaldehyde solution (BioVitrum, St.Petersburg, Russia) for 10 min at room temperature and washed three times with FSB (PanEco, Moscow, Russia). Then, they were treated with a 0.05% Triton-X100 solution (PanEco, Moscow, Russia) for 10 min at room temperature, followed by washing three times in FSB.

To block non-specific antibody binding, cells were treated with a 10% solution of secondary antibody donor serum (goat serum) for 30 min at room temperature. Immediately after the blocking step, cells were incubated with primary human monoclonal antibodies to vinculin (Sigma Aldrich solutions, Merck SA, Darmstadt, Germany) at a dilution of 1:200 in a humid chamber, which was carried out overnight at +4 °C.

After washing the cells from the primary antibody solution in FSB, the Alexa Fluor 488 Goat anti-mouse secondary antibody (Thermo Fisher Scientific, Life Technologies, Carlsbad, CA, USA) was used at a dilution of 1:1000. The cells were incubated for 1.5 h at room temperature in the dark.

Rhodamine-Falloidin (Thermo Fisher Scientific, Life Technologies, Carlsbad, CA, USA) at a working dilution of 1:1000 was used to stain the cytoskeleton of cells when cultured on samples; it was incubated for 1.5 h at room temperature in the dark, according to the manufacturer’s recommendations. After cells were washed to remove unbound dye, nuclei were stained with DAPI (Thermo Fisher Scientific, Life Technologies, Carlsbad, CA, USA).

Images of MG-63 human osteosarcoma cells, labelled with primary antibodies against vinculin and secondary antibodies conjugated with Alexa Fluor 488 and DAPI, were acquired on an A1R confocal system mounted on a Nikon Ti microscope (Nikon, Japan). Aqueous immersion and a Nikon CFI Plan Fluor 20x MImm DIC N2× 0.75 NA objective (Nikon, Tokyo, Japan) were used for imaging. Excitation/emission wavelengths were ex = 405 nm/em = 450 (50) nm (DAPI) and ex = 488 nm/em = 525 (50) nm (FITC, Alexa Fluor 488). Images were processed and counted using NIS Elements ver.4.51 software (Nikon, Tokyo, Japan).

Five fields of view containing an average of 200 cells after 24 h and 72 h of culturing were counted for each term, respectively.

#### 4.7.5. Cytocompatibility Study of MCPC Samples via Direct Contact Method

In vitro cytocompatibility studies of the obtained MCPC samples were performed via the direct contact of the MG-63 cells with the surface of the tested MCPC samples. Sterile samples of the materials were placed in 48-well culture plates (SPL, Gyenggi-do, Korea) (four samples of each type for each term of the experiment: three of them for cytocompatibility assessment; one for the optical blank), and a cell suspension was added at a concentration of 10.0 × 10^3^ cells per well (seeding density of −13 × 10^3^ cells/cm^2^) in the volume of 1 mL CGM. Cultures were run for 1, 3, 7, and 10 days with regular (twice weekly) medium changes. Wells with cells on polystyrene culture plastic served as controls. At the end of the cultivation, cell viability was estimated via the MTT assay according to the above formula.

A sample of material was considered cytocompatible if the PVC value was ≥70%.

#### 4.7.6. Investigation of the Ability of MCPC Samples to Support Osteogenic Differentiation of BM hMSCs

To evaluate the plasticity of BM hMSCs in the osteogenic direction under the conditions of their cultivation on non-toxic MCPC samples, cells were cultured in CGM in 6-well plates (Corning Inc., Corning, NY, USA) at an initial seeding density of 50.0 × 103 cells/cm^2^. To induce osteogenic differentiation when the culture reached the pre-confluent monolayer state (7–9 days of the experiment), the CGM in the wells was changed to a special induction medium (IM) (Differentiation Basal Medium and MesencultTM Differentiation 5x Supplement (Stemcell Technologies, Vancouver, BC, Canada)). The expression of osteogenic differentiation genes was determined via real-time polymerase chain reaction (real-time PCR) for 14 days after the start of differentiation.

According to the experimental design, the following procedures were carried out in the groups:

1. BM hMSCs were cultured on plastic in CGM (control) (BM hMSCs+CGM).

2. BM hMSCs were cultured on plastic in a special induction (osteogenic) medium (BM hMSCs+IM).

3. BM hMSCs were cultured in CGM on the MCPC1 sample (BM hMSCs+Cem1+CGM).

4. BM hMSCs were cultured in a special induction medium on the MCPC1 sample (BM hMSCs+Cem1+ IM).

5. BM hMSCs were cultured in CGM on the MCPC2 sample (BM hMSCs+Cem 2+CGM).

6. BMh MSCs were cultured in special induction medium on the MCPC2 sample (BM hMSCs+Cem2+IM).

7. BM hMSCs were cultured in CGM on the MCPC3 sample (BM hMSCs+Cem 3+CGM).

8. BM hMSCs were cultured in a special induction medium on the MCPC3 sample (BM hMSCs +Cem3+IM).

9. BM hMSCs were cultured in CGM on the MCPC4 sample (BM hMSCs +Cem4+CGM).

10. BM hMSCs were cultured in a special induction medium on the MCPC4 sample (BM hMSCs +Cem4+CGM).

#### Gene Expression Analysis

The changes in the expression levels of osteogenic differentiation marker genes in cells were assessed using real-time PCR. On the 14th day of cell growth in preconfluent conditions, the following genes were analysed: RUNX2, ALPL, and SP7 (Table 3). The expression of the above-mentioned genes was normalised using the glyceraldehyde-3-phosphate dehydrogenase gene (“housekeeping” gene, GAPDH). The total RNA pool was isolated via phenol-chloroform extraction using a Qiazol lysis buffer (Qiagen, Hilden, Germany), followed by purification on RNeasy Mini Kit columns (Qiagen, Hilden, Germany). The amount of RNA in the samples was measured using a NanoDrop ND-2000 spectrophotometer (Thermo Scientific, Waltham, MA, USA). Reverse transcription was performed using the MMLV RT kit (Evrogen, Moscow, Russia) and Random (dN)10-primer. qPCRmix-HS SYBR with SYBR Green I intercalating dye (Evrogen, Moscow, Russia) and a DTlite detecting amplifier (DNA-Technology, Moscow, Russia) were used for real-time PCR assays. The amplification program included a “hot start” (94 °C, 10 min), followed by 50 cycles of template denaturation (94 °C, 20 s), primer annealing (64 °C, 10 s), and amplicon elongation (72 °C, 15 s). All samples were analysed in triplicate. The expression of the gene of interest was normalised to GAPDH [89] using the ΔΔCt method. As a result, the fold change (FC) in the expression levels of the studied genes in the test and control groups was calculated. The control comprised non-treated MSCs incubated under standard conditions (CGM). The synthesis of oligonucleotide sequences of the primers of the target and normalisation gene was carried out by Evrogen (Moscow, Russia). The sequences of primers for marker genes were designed using the NCBI Primer-BLAST tool (www.ncbi.nlm.nih.gov/tools/primer-blast, accessed on 1 June 2025); the sequences are shown in Table 1.

The determination of alkaline phosphatase during the differentiation of hBM MSCs into osteoblasts was carried out via the immunocytochemical method.

In the process of induced osteogenesis, the activity of alkaline phosphatase (qualitative analysis) in the cytoplasm was determined using an EZstain Alkaline Phosphatase Staining kit (CellCulture, HIMEDIA, Mumbai, India) according to the manufacturer’s protocol. For the test, fixed preparations (citrate-acetone-formaldehyde fixing solution—30 s at room temperature) were incubated at room temperature for 30 min in a solution containing naphthol AS-BI phosphate and a freshly prepared diazonium salt solution (the latter was prepared by mixing a sodium nitrite solution with a red-violet FRV solution, adjusted to a pH of 9.5 using 2-amino-2-methyl-1,3-propanediol). The resulting red-purple colour indicates areas of alkaline phosphorus activity.

#### 4.7.7. Statistical Processing of Results

The results were processed using standard methods of variance statistics, which were applied in Microsoft Excel 2000. The reliability of differences was assessed using the parametric Student’s t-criterion. Differences at *p* < 0.05 were considered statistically significant.

## 5. Conclusions

Creating a new injectable bone cement with neutral pH during setting, sufficient strength, and controlled magnesium release—which contributes to the improvement of cytocompatibility and supports the morphogenetic potential of human MSCs in vitro—remains a challenge. We demonstrated that cement powders based on Mg-Wt and St phases in the presence of MgO and cement liquid based on NaH_2_PO_4_ are promising systems for the creation of injectable bioactive bone cement. The introduction of NaHA improved the injectability of MCPCs by increasing viscosity and cement cohesion, and it slightly increased the setting time, making the performance of MCPCs suitable for medical practice. The magnesium content in MCPCs improves their compressive strength and biological characteristics. The release dynamics of Mg^2+^ ions into the medium do not prevent MG-63 cell adhesion or active proliferation on the cement surface; instead, they promote the osteogenic differentiation of donor MSCs, as evidenced by the expression profile of a number of key osteogenesis genes and the deposition of alkaline phosphatase in vitro. The combined physicochemical and biological properties of the developed cement compositions characterise them as bioactive and cytocompatible, making them promising biomaterials for bone defect reconstruction.

## Figures and Tables

**Table 1:** Phase composition according to XRD data, particle size distribution according to laser diffraction measurement, and elemental composition of cement powders according to ICP results.

Cement Powder	Phase Composition			Particle Size Distribution (μm)			Content, wt.% (ICP)			(Ca + Mg)/P
	Mg-Wt	St	MgO	D10	D50	D90	Ca	Mg	P	
20 mol.% Mg	84	0	16	0.8	5.8	18.6	18.8	15.8	20.4	1.67
60 mol.%Mg	0	90	10	1.2	11.7	57.6	18.3	15.8	20.5	1.66

**Table 2:** Viscosity and surface tension of cement liquids.

Cement Liquid	Concentration of Polymer NaHA, %	Viscosity, mPa·Sec		Surface Tension, mN/m
		50 rmp	200 rpm	
A	-	2.4 ± 0.5	6.00 ± 0.5	79.5
A+1%NaHA	1.00	12 ± 0.5	24.5 ± 0.5	83.0

**Table 3:** Gene symbols and sequences of primers for the analysed and normalised genes.

№	Gene Symbol	Encoded Protein	F and R Primer Sequences 5′-3′
1	*RUNX2*	Runt-associated transcription factor 2 is one of the key regulators of osteoblastic cells	*F: tca-acg-atc-tga-gat-ttg-tgg-g* *R: ggg-gag-gat-ttg-tga-aga-cgg*
2	*SP7*	Osterix is a transcription factor involved in the differentiation of mesenchymal progenitors into osteoblasts and osteocytes	*F: ccc-acc-tac-cca-tct-gac-tt* *R: gct-gcc-cac-tat-ttc-cca-ct*
3	*ALPL*	Alkaline phosphatase is a membrane-bound glycosylated enzyme involved in matrix mineralisation	*F: acc-acc-acg-aga-gtg-aac-ca* *R: cgt-tgt-ctg-agt-acc-agt-ccc*
4	*GAPDH*	Glyceraldehyde-3-phosphate dehydrogenase, housekeeping gene	*F: gaa-ggt-gaa-ggt-cgg-agt-c* *R: gaa-gat-ggt-gat-ggg-att-tc*

## Data Availability

Data is contained within the article.
